# Bilateral skull fracture with massive epidural hematoma secondary to pin-type head fixation in a pediatric patient: Case report and review of the literature

**DOI:** 10.1016/j.ijscr.2019.07.079

**Published:** 2019-08-13

**Authors:** M. Arifin Parenrengi, Fatkhul Adhiatmadja, Muhammad Reza Arifianto, Tedy Apriawan, Asra Al Fauzi, Franco Servadei

**Affiliations:** a Department of Neurosurgery, Faculty of Medicine, Universitas Airlangga, Dr. Soetomo Academic Medical Center Hospital, Surabaya, Indonesia; b Department of Neurosurgery, Universitas Airlangga Teaching Hospital, Surabaya, Indonesia; c Department of Neurosurgery, Humanitas University-Research Institute, Milan, Italy

**Keywords:** CT, computed tomography, GCS, Glasgow Coma Scale, EVD, extra-ventricular drainage, CSF, cerebro spinal fluid, AED, anti-epilepsy drugs, Skull fracture, Epidural hematoma, Head fixation, Pediatric patient

## Abstract

**Introduction:**

A head fixation device containing pins is common equipment used in neurosurgical procedures. Previous reports analysed some of the complications associated with the application of this device for adults and children, even the indications for the use in pediatric are not well defined.

**Case presentation:**

An 11-year-old girl diagnosed with non-communicating hydrocephalus, caused by posterior fossa tumor. During the surgery, complications were found in the form of acute epidural hematoma due to head fixation pins. So, the operation was stopped. Emergent CT scan was carried out and showed a bilateral skull fracture and a massive right-sided epidural hematoma. An emergency craniotomy for clot removal was performed and five days later, a second surgery was carried out uneventfully for the residual tumor. The patient fully recovered after the second surgery.

**Discussion:**

Complications due to the use of a pin head fixation are easier to occur in pediatric patients, because the bones are thinner and need more carefull strategy when pinning. With promp identification of any complications and earlier treatment, the good outcome will be achieved. We compared our case report with published literature in order to suggest the way to prevent this complication.

**Conclusion:**

Skull fractures and associated epidural hematomas in pediatric patients need to be considered as possible complications of the pin-type head fixation application. The head fixation devices in pediatric need to be used with great caution and knowing the risk factors, safe technique for application and management of complications will prevent worse outcome.

## Highlights

• Epidural hematoma in pediatric cases due to head fixation pins are rarely reported.
• The use of pin-type head fixation in pediatric has several different risk factors compared to adult patients.
• By knowing pre-operative risk factors and early treatment, the outcome will be better.

## 1 Introduction

For an ideal and safe positioning of the patient during surgery, the pin-type fixation is widely used to keep the patient's head and neck in a stable position and it is also mandatory for optimal accuracy [[1], [2], [3], [4], [5]]. Complications directly related to the application are rare but potentially life-threatening. There are some reported cases of complication in both adult and children. The safe use in children has not been clearly defined and the device is therefore generally not recommended under the age of 5 years. However, the safety and guidelines for application in older children also are not entirely known [1,4]. The case of an unusual complication in an 11 years old girl who developed a bilateral depressed skull fracture and an epidural hematoma due to the usage of the Mayfield fixation device is reported. Additionally, we review the published risk factors, management, outcome and propose recommendations for the application in pediatric patient to avoid unnecessary complications. The work has been reported in line with the SCARE criteria [6]. Written informed consent was obtained from the patient’s family for publication of this case report and accompanying images.

## 2 Presentation of case

### 2.1 History and initial examination

**Fig. 1:**
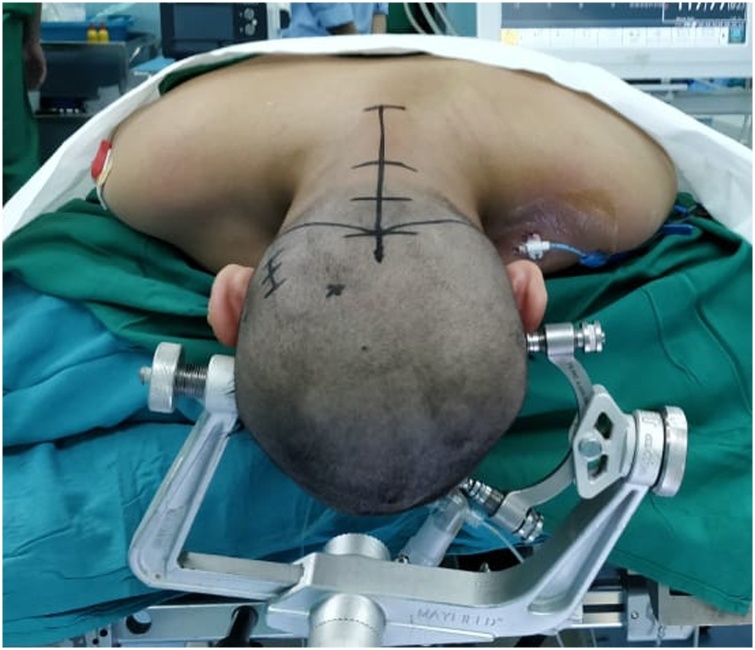
Mayfield® three-pin skull clamp is applied to the patient’s head with prone position.

An 11 years old girl, with a gradual decrease of consciousness for three days before admission presented to our emergency department. A year before because of visual impairment she was diagnosed with non-communicating hydrocephalus due to the cerebellar tumor in another remote hospital. At that time, the patient's family refused to undergo surgery and never took a further follow up. On the physical examination, GCS was 14 out of 15 with total blindness on both eyes, no cerebellar signs, no motor, and sensory deficit. The CT scan revealed a posterior fossa lesion which caused non-communicating hydrocephalus (Fig. 1).

### 2.2 Operative technique

**Fig. 2:**
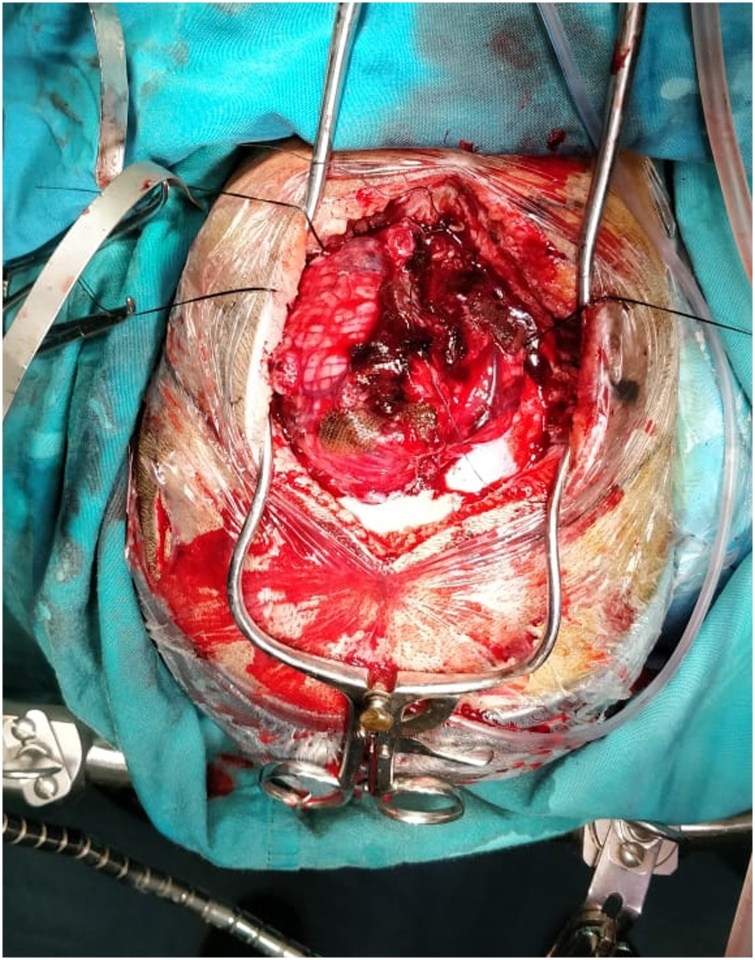
During the operation, the cerebellum has bulged.

**Fig. 3:**
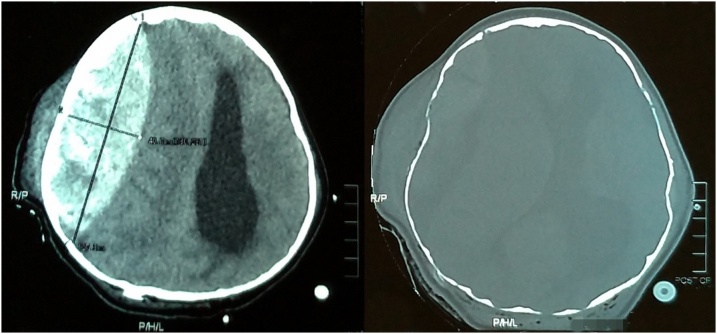
An emergent CT head evaluation shows bilateral depressed skull fracture and a massive epidural hematoma with 40 mm thickness causing 10 mm midline shift to the left.

The girl underwent an emergency craniotomy for tumor removal. She was placed in 3-point pin fixation using the Mayfield device in a prone position. After stabilizing the head and neck, the chief resident performed the procedure to penetrate the skull using pins no more than 40 pounds. The operation began with the insertion of extra-ventricular drainage (EVD) on the right occipital and followed by a suboccipital craniotomy. Prior to the opening of the dura, the ventricular tapping was opened with approximately 10cc of the CSF was drained and this maneuver was controlled, drained out again when needed. Using the midline telovelar approach, the tumor was reached and a piecemeal resection was started, after a partial removal of the tumor, the exposed cerebellum suddenly began to swell usually through the bone flap despite intermittent CSF drainage (Fig. 2), we realized that there was some complication and the operation was eventually discontinued after hemostasis of the tumoral area. A right side anisocoria was noted and an emergency CT scan was performed while the patient was still under anesthesia, The head CT scan showed a massive subgaleal hematoma and a bilateral depressed fracture at the pin-site with a large right-sided epidural hematoma of over 40 mm thickness causing a 10 mm midline shift to the left (Fig. 3). An emergency craniotomy for the evacuation of the hematoma was conducted. During the surgery, it was noted that the bone was abnormally thin for the patient’s age. A laceration of the dura under the depressed fracture was also demonstrated.

### 2.3 Post-operative course

Post-operatively, the patient gained full consciousness without neurological deficit. Five days later a second surgery for the remaining tumor was done uneventfully. A subsequent pathology report confirmed the diagnosis of medulloblastoma (Table 1).

**Table 1:** Summary of the literature review of skull fracture with or without epidural hematoma cases secondary to pin-type head fixation in children.

Author (year)	Age, years, Sex	Diagnosis	HC	Surgical procedure	Release CSF	Position	Type of Device	Complications	On set of complication	Treatment of complication	Outcome	Risk Factors
Pang et al. [7]	7 F	Juvenile Cerebellar Astrocytoma	+	SCo - TR	–	Sitting Position	Three pin head fixation	Depressed skull fracture with scalp laceration and CSF leak Air embolism Subdural bifrontal pneumochepalus	During surgery	Air suction via intracardiac catheter and closing of the scalp and dural laceration	VP shunt infection with significant cerebellar deficits	Chronic hidrocephalus Thin calvarial bone
Baerts et al. [8]	10 F	Frontal Lobe Glioma	−	RFtC - TR	–	Supine	Mayfield	Impressed skull fracture + Supratentorial epidural hematoma	Delayed, ten days after surgery	Small craniotomy and clot evacuation	Favorable	Chronic high intracranial pressure
Lee et al. [4]	8 F	Medulloblastoma	+	SCo	VP shunt	Prone	Mayfield	Depressed skull fracture + Dural laceration with small cerebral contussion	Immediate prior to surgical procedure	Elevation of the bone fragment & exploration of duramater	Favorable	Hidrocephalus Thin calvarial bone
	4 M	Hypothalamic glioma		FC - TR	–	Supine	Sugita	Depressed skull fracture + Dural laceration	Immediate prior to surgical procedure	Elevation of the bone fragment & exploration of duramater	Favorable	no data
	3 F	Brainstem glioma		SCo - TR	–	Prone	Mayfield	Depressed skull fracture + Dural laceration	Immediate prior to surgical procedure	Elevation of the bone fragment & exploration of duramater	Favorable	no data
	8 M	Brainstem glioma	+	SCo - TR	VP shunt	Prone	Sugita	Depressed skull fracture + Dural laceration	Delayed postoperative	No surgical treatment	Favorable	no data
	5 M	Hypothalamic glioma		FC – TR	–	Supine	Sugita	Depressed skull fracture + Dural laceration with cortical laceration	Immediate prior to surgical procedure	Elevation of the bone fragment & exploration of duramater	Favorable	no data
Medina et al. [9]	13 M	Supratentorial cystic lesion	−	RPC, TP and removed the lesion	–	Supine	Mayfield	Supratentorial epidural hematoma	During the surgery	EC and CR	Severe right hemiparesis and aphasia	Abnormal thinness of the skull due to chronic high intracranial pressure
Tang et al. [10]	15 M	Medulloblastoma	+	SCo + TR	–	Prone	Mayfield	Depressed skull fracture + Supratentorial epidural hematoma	Delayed, seven hours after surgery	EC and CR	Favorable	Chronic hydrocephalus
Yan et al. [11]	15 M	Posterior fossa tumor (choroid plexus paplilloma)	+	SCo + TR	EVD	Prone	Mayfield	Impressed skull fracture + Supratentorial epidural hematoma	Delayed, six hours after surgery	EC and CR	Favorable	Chronic hydrocephalus Thin calvarial bone
Vitali et al. [12]	2,7 M	Ependymoma	+	SCo + TR	–	Prone	Mayfield	Temporal skull fracture + Supratentorial epidural hematoma	Immediately after surgery	Emergency exploratory then craniotomy and CR	Favorable	no data
	2,10 F	Ependymoma	+	SCo + TR	–	Prone	Mayfield	Temporal skull fracture + Small epidural hematoma	During the registration for frameless stereotaxy, prior to surgery	CT	Favorable	no data
	4,3	Medulloblastoma	+	SCo + TR	–	Prone	Mayfield	Temporal skull fracture + Small epidural hematoma	Immediately after surgery	CT	Favorable	no data
	4,9	Pineoblastoma	+	SCo + TR	–	Prone	Mayfield	Temporal skull fracture + Massive epidural hematoma	During surgery	EC and CR	Favorable	no data
	6,6	Medulloblastoma	+	SCo + TR	EVD	Prone	Mayfield	Temporal skull fracture + Large epidural hematoma		EC and CR	Favorable	no data
Martínez-Lage et al. [13]	4 F	Pilocytic cerebellar astrocytoma	+	SCo + TR	EVD	Prone	Mayfield	Depressed skull fracture + Pneumocephalus	Immediately after surgery	CT	Favorable	Chronic hydrocephalus
Poli et al. [14]	7 M	Pilocytic cerebellar astrocytoma		SCo + TR	–	Prone	Mayfield	Depressed skull fracture + Supratentorial epidural hematoma	Immediately after surgery	EC and CR	Favorable	no data
Chen et al. [15]	6 F	Posterior fossa tumor	+	SCo + TR	EVD	Prone	Mayfield	Temporal depressed skull fracture + Epidural hematoma	Immediately after surgery	EC and CR	Favorable	no data
Khrisnan et al. [16]	12 F	Posterior fossa tumor	+	SCo + TR	EVD	Prone	Three pin head fixation	Temporal depressed skull fracture + Epidural hematoma	During surgery	C1-C2 laminectomy EC and CR	Residual ataxia and cerebellar signs	no data
Moutaokil et al. [17]	17 M	Medulloblastoma	+	SCo + TR	VP shunt	Prone	Mayfield	Parietal depressed skull fracture	After surgery	CT	Favorable	Chronic hydocephalus
Present case	11 F	Medulloblastoma	+	SCo + TR	EVD	Prone	Mayfield	Temporal depressed skull fracture + Epidural hematoma	During surgery	EC and CR	Favorable	Chronic hydrocephalus Thin calvarial bone

SCo = Suboccipital Craniotomy; TR = Tumor Removal; EVD = External Ventricular Drain; VP = Ventriculoperitoneal; RPC = Right Parietal Craniotomy; TP = Transcortical Puncture; FC = Frontal Craniotomy; RFtC = Right Fronto-temporal Craniotomy; EC = Emergency Craniotomy; CR = Clot Removal; CT = Conservative Treatment.

## 3 Discussion

The pin-type is a widely used standard head fixation device and various modifications have been developed as it is designed to anchor the outer table of the cranial vault [12,13,[18], [19], [20]]. The commonly used types of pin in our area are the Mayfield and the Sugita devices. The Mayfield system provides a 3-pin head fixation and permits the adjustment of the force with a torque screw up to about 80 pounds [2]. The Sugita system is a semi-circular fixation type with a 4-pin skull fixation and each screw must be screwed separately [2]. Previous reports show that indications for the safe use of the pins in children are not well defined [8,21]. From the literature review, the age in which the pin fixations for cranial surgery can be considered completely safe is not yet made clear [12]. Berry et al. in their study, surveying practices among pediatric neurosurgeons notes that there are no clear-cut guidelines for the use of head fixation in children despite the rare but significant complications that may be involved [1].

Complications related to the use of pin head fixation are quite rare ranging from 0.65% to 1.1% [1,2,12]. PubMed, Google Scholar, and Science Direct searches were implemented using some keyword which is: “Mayfield device”, “head fixation”, “skull fracture”, and “epidural hematoma” to find related journals and articles. Adult data were separated from the pediatric data and used for general analysis but not considered in our particular analysis. The most frequent complications in children are skull fracture and epidural hematoma [2]. The variability in the thickness of the developing cranium is likely the most important factor causing a higher number of complications in children. In a survey among 605 pediatric neurosurgeon about pin complications, 164 responders have reportedly experienced some complications and the two most common complications are depressed skull fracture (59 of 89) (66%) and epidural hematoma (43 of 89) (49%). In our literature review, only 21 cases including the present case were published in the English literature from 1982 until 2018. There were 8 cases of skull fractures only and 13 cases of skull fractures with epidural hematomas. Seventeenth of 21 patients required a separate surgical procedure, with 5 cases (29%) needing exploration and elevation of depressed fracture: Eleven cases (65%) needed a craniotomy and clot removal. In our case, the epidural hematoma was detected intra-operatively whereas some other reports discovered the complication prior to the surgical procedure or even after surgery.

Some authors suggest that the use of the pin-type head fixation should be avoided in children especially in unnecessary cases [12,14,19]. The review showed that most complications were related to the squamous temporal bone which is the thinnest [15]. The other areas to avoid is the frontal sinus and coronal suture [10]. Some authors recommend the avoidance in children under 5 years [12,14,16,20], and another author reports a firm contraindication forchildren under 2 years of age [1,4]. It is, however, important to know that 2 or 5-year-old cut-offs is not based on scientific evidence and this age recommendation may not be a failsafe strategy [12]. Patients with an intracranial pathology which results in a long-standing increased intracranial pressure and chronic hydrocephalus may contribute to thinning of the skull, making it a greater risk of skull fracture [5,16]. Long-term usage and high doses of anti-epilepsy drugs (AED) can cause brittle bones [18]. Most of the pediatric patients with intracranial pathology usually take AED to prevent seizures, but this is not clearly described in the report articles. More than 75% of the cases happened in a prone position with a posterior fossa tumor and hydrocephalus. Some physiological studies may explain this massive bleeding in the prone position as an increase in cerebral venous pressure [22,23].

In the prevention of skull fracture, most authors recommend using a pediatric-sized pin and applying the appropriate pressure in accordance with the age and skull thickness [1,4,12,13]. For a thinner skull, a lesser force is generally recommended [5,24]. Five pounds are used in children between 6 and 12 months, 10 pounds for those between 12 months to 2 years, 20 pounds for 2 to 5 years, and 30 pounds of force for those between 5 to 12 years [1,24]. In our case, we already used an appropriate force no more than 40 pounds but did not predict the risk factor of the high chronic intracranial pressure which can cause thinning of the cranium and an increased volume of the diploe vein. In our case, the only occasion where a bilateral depressed skull fracture occurred, showed the possibility of an error when applying the pins on both sides. In our teaching hospital, it has indeed become a commonplace that those responsible for carrying out this pre-operative procedure are chief residents. In pediatric cases, we recommend that in future pin application should be carried out by experienced pediatric neurosurgeon whenever possible. Naik et al stated that drainage of CSF from the cistern led to the loss of the tamponed effect which aggravates bleeding from the puncture site [25].

Di Rocco has commented on the need to design a safer device that would comply with requirements of the delicate head of the pediatric population [26]. Many modifications of head fixation have been reported. Aoki and Sakai describe using of medical bottle rubber cup over the skull pins as a barrier to the pins penetrating too deeply [1,13,20,27]. Others recommend a simple technique using an adhesive drape, such as U-drape over a horseshoe headrest so as to stabilize the children’s head and provide a rigid immobilization [12,15,28]. Muzumdar et al. describe a method of using plaster of Paris as an interface between the frame and the patient's head in a pediatric stereotactic procedure [3]. Though considered as satisfactory, these modification systems do not eliminate slippage as the clamp is not directly attached to the cranium. Gupta utilizes the modification of the Mayfield system combined with a padded horseshoe headrest because the system enables the pins to be tightened as much as the force is reduced and it would still maintain a rigid position [3,13]. Lee et al. suggest using all six pin sites on the Sugita head fixation system (rather than only four) to decrease the individual pin pressure, immobilize the head, and thus lessen the risk of a depressed fracture [1,4].

The emergency craniotomy and hematoma evacuation were mandatory for life-saving management in cases of the head fixation related complications. In our literature review, there were 13 of 21 cases which reportedly had epidural hematoma complications. There were 4 cases including our cases detected during surgery, 4 cases immediate after surgery and 3 cases detected delayed after surgery. When there is a significant epidural hematoma, an emergency craniotomy should be done prior to proceeding to the initially planned procedure [12]. In present cases, the surgery was abandoned midway in order to get the imaging done and then we proceed to an emergency craniotomy for clot removal.

Considering the outcome in such complications, there was no mortality in all reported cases. In our literature review of 21 cases, there were sequelae in only 3 patients while other 18 cases showed a favorable outcome. Although our patient sustained a massive epidural hematoma with anisocoria which resulted to suspension of the operation, the eventual result after the hematoma evacuation was gratifying.

### 3.1 Proposed suggestions to avoid complications

#### 3.1.1 Preoperative

1 Avoiding the usage of pin-type head fixations if not really necessary
2 Examine carefully the pre-operative CT scan to determine the cranial suture and the thickness of the bone.
3 An alternative rigid fixation using the combination of a pin headrest and a padded head holder especially for infants and young children

#### 3.1.2 Technique of applications

1 Using child dedicated pins
2 Avoiding the weak zones of the skull as much as possible
3 Installation of pins must be done by a senior or pediatric neurosurgeon
4 A slow and less CSF drainage to prevent the loss of brain tamponade effect
5 Disengaging the pins when patients are in a horizontal position to avoid the risk of air embolism

#### 3.1.3 Management of complications

1 Always be aware of possible complications: when inserting the pins, during operation, immediately after surgery and during post-operative in the ward
2 In a case of suspected complications, the procedure should be stopped immediately and a CT scan diagnostic performed.
3 Emergency craniotomy and clot removal must be done for life-saving.

## 4 Conclusion

Skull fractures and associated epidural hematomas in children need to be considered as possible complications of the pin-type head fixation application with some of them severe and life-threatening. The head fixation devices in the pediatric population need to be used with great caution and the only way to avoid complications is to avoiding using pins on these patients. In cases of necessary use of the pins for rigid immobilization, every neurosurgeon should be aware of the risk factors, type of device, safe technique for application and management of complications when it occurs. Anyway, prompt identification and proper management of this rare complication usually result in good outcome as in our case.

## Sources of funding

This research did not receive any specific grant from funding agencies in the public, commercial, or not-for-profit sectors.

## Ethical approval

This is a case report; therefore it did not require ethical approval from ethics committee. However, we have got permission from her parents to publish her data.

## Consent

The study was conducted with the human subject’s understanding and consent.

## Author contribution

Muhammad Arifin Parenrengi: Writing the paper, design and data collection, data analysis and interpretation.

Fatkhul Adhiatmadja: Design and data collection, data analysis and interpretation.

Muhammad Reza Arifianto: Design and data collection, data analysis and interpretation.

Teddy Apriawan: Data analysis and interpretation.

Asra Al Fauzi: Data analysis and interpretation.

Franco Servadei: Data analysis and interpretation.

## Registration of research studies

Not available.

## Guarantor

Asra Al Fauzi.

## Declaration of Competing Interest

The authors report no conflicts of interest.
